# Leveraging digital health technologies for efficiency in general practice

**DOI:** 10.3399/BJGPO.2025.0200

**Published:** 2026-02-11

**Authors:** Kayode Philip Fadahunsi, Katie Massey, Azeem Majeed

**Affiliations:** 1 Department of Primary Care and Public Health, Imperial College London, London, UK; 2 Lincolnshire Training Hub, Lincoln, UK

**Keywords:** Efficiency, Digital health technology, General practitioners, Primary healthcare

## Introduction

General practice in the UK is facing unprecedented pressures, including rising patient demand, complex multimorbidity, and workforce shortages.^1^ GPs spend a significant proportion of their time on administrative tasks such as documentation, correspondence handling, and patient communications, leaving less time for direct patient care.^2^ Administrative burdens not only contribute to inefficiency but also lead to professional burnout, dissatisfaction, and early departure from the workforce.^3^ Administrative inefficiency is therefore not merely an inconvenience; it directly affects the quality of patient care and the efficiency and productivity of health services.^3^ Addressing this challenge should therefore be a strategic priority for the NHS and the Department of Health and Social Care.

Digital health technologies, if appropriately harnessed, offer opportunities to streamline workflows, reduce duplication, and release time for patient-facing work.^4^ However, the promise of digital tools must be balanced against concerns about usability, data governance, and unintended consequences, such as digital exclusion or additional workload from poorly implemented systems. The 'paperless NHS' vision,^5^ first articulated over two decades ago, remains only partially realised, with incompatible electronic systems and inconsistent use of templates contributing to variation in practice. In this commentary, we reflect on the practical experience from a ‘New to Practice Fellowship’ project, where the fellow (KPF) explored the use of tools within SystmOne and AccuRx to enhance administrative efficiency in his role as a salaried GP in a practice with a list size of over 10 000 patients.^6^

SystmOne and EMIS Web are the dominant electronic health record systems in general practice in England.^7^ While they offer a wide array of functions, many clinicians are unaware of, or underuse, time-saving features such as presets and macros. Similarly, AccuRx, initially adopted during the COVID-19 pandemic to enable secure SMS and video communication, has developed into a broader digital toolkit that can reduce administrative overheads if used effectively.^8^ The New to Practice Fellowship project involved the pragmatic use of SystmOne preset tasks and AccuRx message templates as digital communication pathways to meaningfully reduce GP workload and improve patient experience.^6^

### Preset tasks in SystmOne

Preset tasks represent a relatively underutilised feature within SystmOne that can significantly enhance efficiency. Traditionally, GPs and practice staff manually generate tasks for referrals, follow-ups, or repeat processes. By designing standardised preset tasks, individual GPs can reduce administrative burdens by removing the need to repeatedly type the same instructions. They also enhance safety by ensuring that essential elements (for example, specific blood tests) are not omitted. Preset tasks can also be useful while reviewing blood test results and clinical letters, where actions to be undertaken can be sent to relevant staff as tasks. Our experience during the New to Practice Fellowship project^6^ was that adoption of preset tasks reduced task creation time, freeing the clinician to focus on decision-making rather than administrative transcription. In addition, task standardisation can support cross-cover within practices: locums or new staff members can copy preset tasks from existing staff. An example of a preset task in our project is included as Table 1.

**Table 1.** An example of a preset task

CFS Clinic Bloods: FBC. ESR, CRP, U&E, LFT, Urinalysis, TFT, Random Glucose, Endomysium, Transglutaminase, Antigliadin IgG & IgA antibodies, Vitamin B12, Folic acid, ANA, dsDNA, ENA, Ferritin, Calcium and Phosphate, Serum Creatinine, Creatinine Kinase, Vitamin D.

ANA = antinuclear antibody. CFS = chronic fatigue syndrome. CRP = C-reactive protein. dsDNA = double-stranded deoxyribonucleic acid. ENA = extractable nuclear antigens. ESR = erythrocyte sedimentation rate. FBC = full blood count. LFT = liver function test. TFT = thyroid function tests. U&E = urea and electrolytes.

### AccuRx message templates

Digital communication with patients is increasingly routine. SMS and secure messaging offer quick, traceable alternatives to postal letters and telephone calls. AccuRx has become embedded in many practices as a platform for patient communication, appointment reminders, and follow-up instructions. However, without templates, clinicians often type repetitive messages, increasing both administrative time during consultations and the risk of inconsistency. Our experience with message templates, for example, to provide investigation results, showed that they streamline communication, ensure consistent wording, and allow safety netting, as shown in Table 2. Furthermore, they contribute to patient empowerment: consistent, clear digital communication helps patients better understand their care plan, reducing the likelihood of unnecessary follow-up contacts.

**Table 2.** An example of an AccuRx message template

I am glad to inform you that your ultrasound scan was fine. Hope this is reassuring. If any concerns or your symptoms persist, please book an appointment to discuss further.

### Implementation strategies

Adopting digital tools is challenging. Resistance often stems from lack of awareness, variable digital literacy among staff, or concerns about safety and governance. For successful implementation, we propose integrating digital efficiency into induction programmes for new staff, ensuring familiarity with preset tasks and message templates from the outset. Peer-led training sessions within practice meetings could further raise awareness of their utility. In addition, involving clinicians, administrators, and patients in the design of templates will ensure they are fit for purpose and use clear, patient-centred language. Shared template libraries could be developed across Primary Care Networks (PCNs) to reduce duplication of effort. Periodic reviews and updates are required to maintain accuracy and compliance with NICE or local guidance. Regular audit of usage and impact through quantitative (for example, time saved, reduced duplication) and qualitative (staff and patient feedback) approaches will help to ensure that efficiency for clinicians does not compromise communication quality for patients. Future research should rigorously evaluate the impact of administrative digital tools, using mixed-methods approaches to capture both measurable efficiency gains and nuanced effects on professional identity, continuity of care, and equity.

### Wider implications for general practice

The incremental efficiency gains from digital tools such as SystmOne presets and AccuRx templates, when aggregated across a practice, can reduce administrative workload considerably. If each GP saves just 10 minutes per session, the collective impact across a practice population could equate to dozens of hours of reclaimed clinical time each week. This additional capacity could be redirected to complex patient care, continuity, or preventive health initiatives. Moreover, efficiency tools also contribute to workforce sustainability. Retention of early-career GPs is particularly sensitive to workload and burnout.^3^ By reducing 'friction costs’ in daily practice, digital health can support job satisfaction and wellbeing, aligning with the NHS England fellowship programme’s aims of supporting career development and resilience.^9^ At a system level, streamlined communication enhances care coordination across primary and secondary care, especially if templates are aligned with referral pathways and shared care agreements.

### Limitations and risks

Despite the benefits of these digital tools, it is important to be aware of potential limitations. For example, over-reliance on templates may lead to de-personalised communication. Tweaking the message to the patient’s context at the point of use, when necessary, could prevent this. Furthermore, digital communication assumes patient access to mobile devices and health literacy; those without these may be at risk of exclusion. To reduce digital exclusion, patients who choose to opt out should be offered suitable alternatives such as letters or telephone calls. Practices should also proactively identify patients at risk of digital exclusion, such as older or non-English-speaking individuals, to ensure equitable access to care. Data security remains paramount, requiring adherence to GDPR and Caldicott principles.

## Conclusion

Administrative burdens are a major contributor to inefficiency and burnout in UK general practice.^3^ Digital tools, such as SystmOne preset tasks and AccuRx message templates, offer pragmatic, implementable solutions to streamline workflows and improve both clinician and patient experience. While digital tool adoption is not a panacea, when underpinned by thoughtful design, governance, and training, these tools can reclaim valuable clinical time and support the sustainability of the general practice workforce. The challenge ahead lies in embedding these practices consistently, scaling them across networks, and ensuring that the benefits are equitably distributed across all patient groups. In an era where general practice is under immense strain, small steps towards administrative efficiency may yield disproportionately large dividends in clinician wellbeing and patient care. NHS policymakers and primary care leaders should prioritise investment in training and shared template libraries to scale these efficiencies nationwide.

## Data

N/A
